# Discovery of a multispecies shark aggregation and parturition area in the Ba Estuary, Fiji Islands

**DOI:** 10.1002/ece3.4230

**Published:** 2018-06-25

**Authors:** Tom Vierus, Stefan Gehrig, Juerg M. Brunnschweiler, Kerstin Glaus, Martin Zimmer, Amandine D. Marie, Ciro Rico

**Affiliations:** ^1^ School of Marine Studies Faculty of Science, Technology and Environment The University of the South Pacific Laucala Suva Fiji; ^2^ Leibniz Centre for Tropical Marine Research (ZMT) Bremen Germany; ^3^ Department of Biosciences University of Exeter Penryn Campus UK; ^4^ Independent Researcher Zurich Switzerland; ^5^ Faculty of Biology/Chemistry University of Bremen Bremen Germany; ^6^ Department of Wetland Ecology Estación Biológica de Doñana, Consejo Superior de Investigaciones Científicas (EBD, CSIC) Sevilla Spain

**Keywords:** blacktip sharks, elasmobranchs, hammerhead sharks, neonates, shark bycatch, young‐of‐the‐year sharks

## Abstract

Population declines in shark species have been reported on local and global scales, with overfishing, habitat destruction and climate change posing severe threats. The lack of species‐specific baseline data on ecology and distribution of many sharks, however, makes conservation measures challenging. Here, we present a fisheries‐independent shark survey from the Fiji Islands, where scientific knowledge on locally occurring elasmobranchs is largely still lacking despite the location's role as a shark hotspot in the Pacific. Juvenile shark abundance in the fishing grounds of the Ba Estuary (north‐western Viti Levu) was assessed with a gillnet‐ and longline‐based survey from December 2015 to April 2016. A total of 103 juvenile sharks identified as blacktip *Carcharhinus limbatus* (*n* = 57), scalloped hammerhead *Sphyrna lewini* (*n* = 35), and great hammerhead *Sphyrna mokarran* (*n* = 11) sharks were captured, tagged, and released. The condition of umbilical scars (68% open or semihealed), mean sizes of individuals (±*SD*) (*C. limbatus:* 66.5 ± 3.8 cm, *S. lewini:* 51.8 ± 4.8 cm, *S. mokarran* 77.4 ± 2.8 cm), and the presence of these species over recent years (based on fishermen interviews), suggest that the Ba Estuary area is a critical habitat for multiple species that are classified as “Near Threatened” or “Endangered.” Specifically, the area likely acts as a parturition ground over the studied period, and potentially as a subsequent nursery area. We identified subareas of high abundance and found that temperature, salinity and depth acted as small‐scale environmental drivers of shark abundance. The data suggests a tendency for species‐specific spatial use, both horizontally (i.e., between sampling areas) and vertically (i.e., across the water column). These results enhance the understanding of shark ecology in Fiji and provide a scientific basis for the implementation of local conservation strategies that contribute to the protection of these threatened species.

## 1 INTRODUCTION

Chondrichthyan fishes (sharks, rays, and chimaeras) are under increasing pressure from human activities such as fishing and habitat degradation (Dulvy et al., 2014; Heupel & Simpfendorfer, 2011; Jennings, Gruber, Franks, Kessel, & Robertson, 2008). Often in combination, these activities particularly affect species that regularly use inshore regions and estuaries during various stages of their life‐history. Nearshore environments are important for feeding, mating, parturition, and energy conservation, and serve as nursery areas for many shark species (Bansemer & Bennett, 2009; Barnett, Stevens, Frusher, & Semmens, 2010; Carlisle & Starr, 2009; Curtis, Parkyn, & Burgess, 2013; Harasti, Lee, Bruce, Gallen, & Bradford, 2017; Heupel, Carlson, & Simpfendorfer, 2007; Ubeda, Simpfendorfer, & Heupel, 2009; Yeiser, Heupel, & Simpfendorfer, 2008). Hence, it is essential that these habitats are effectively managed to protect sharks from detrimental anthropogenic impacts, especially in the face of direct and indirect effects of climate change, which will increase vulnerability of coastal shark species (Chin, Kyne, Walker, & McAuley, 2010). Given their proposed ecological function as a keystone predator in many aquatic communities (Heupel, Knip, Simpfendorfer, & Dulvy, 2014), sharks urgently require scientifically informed management measures, not only to conserve biodiversity on a larger scale but also to maintain local ecosystem services. Due to typically slow growth, late maturity, and low fecundity of individuals, shark populations are supposed to be less resilient to disturbances than other fish stocks (Musick, Burgess, Cailliet, Camhi, & Fordham, 2000; Smith, Au, & Show, 1998).

The establishment of marine protected areas is a popular conservation strategy that has been shown to support shark populations, or at least to mitigate detrimental human activities in critical nearshore areas (Aburto‐Oropeza et al., 2011; Henderson, Jourdan, & Bell, 2016; Knip, Heupel, & Simpfendorfer, 2012). Selecting appropriate locations, however, requires the identification of shark habitats, which may not only differ between species and across regions, but may also shift with the requirements of certain life‐history stages (Grubbs, 2010; Ward‐Paige, 2014). Such basic information is often scarce, particularly in many rural and developing coastal areas. One example of such a region are the Fiji Islands. At least 17 shark species are known to occur in Fijian waters (Seeto & Baldwin, 2010), but generally little is known about exactly where different species concentrate, and how and when they make use of the available habitats. Using data collected from dive operators, citizen scientists and local fishermen, an increasingly clear picture of shark species abundance throughout Fiji is emerging (Brunnschweiler, Abrantes, & Barnett, 2014; Glaus, Adrian‐Kalchhauser, Burkhardt‐Holm, White, & Brunnschweiler, 2015; Rasalato, Maginnity, & Brunnschweiler, 2010; Ward‐Paige, 2014). Specific locations with confirmed species occurrence in the scientific literature are only available for Viti Levu (Brown, Seeto, Lal, & Miller, 2016; Brunnschweiler & Earle, 2006; Cardeñosa, Glaus, & Brunnschweiler, 2017; Marie, Miller, Cawich, Piovano, & Rico, 2017) and Vanua Levu (Goetze & Fullwood, 2013), the two largest islands of Fiji. In the former case, this has led to the establishment of the Shark Reef Marine Reserve, Fiji's first national marine park, and the Fiji Shark Corridor which comprises approximately 30 miles of coastline (Brunnschweiler, 2010).

The main threat to sharks in Fijian waters is their frequent occurrence in the bycatch of artisanal and subsistence fisheries in the inshore fishing grounds (Glaus et al., 2015). This includes not only coastal waters but also rivers and river deltas, as shown by Rasalato et al. (2010) who collected interview‐based evidence of shark occurrences in all of Fiji's rivers. Ecological studies recently confirmed the usage of riverine and estuarine habitats by juvenile sharks in both the Navua and Rewa River in southern Viti Levu (Brown et al., 2016; Cardeñosa et al., 2017). There is currently no systematic data on shark occurrence in estuaries on the northern coast of Fiji's main island Viti Levu.

Thus, in this study we investigate for the first time the Ba Estuary on the northern coast of Viti Levu and aim to assess (a) the composition of shark species occurring in the area, (b) their abundance and life‐history stages, (c) spatiotemporal differences in habitat use over 4 months, and (d) environmental drivers of abundance. Furthermore, through semi‐structured interviews with local fishermen, we provide socio‐economic context that also explores community support for potential management options.

## 2 MATERIAL AND METHODS

### 2.1 Study site

**Figure 1:**
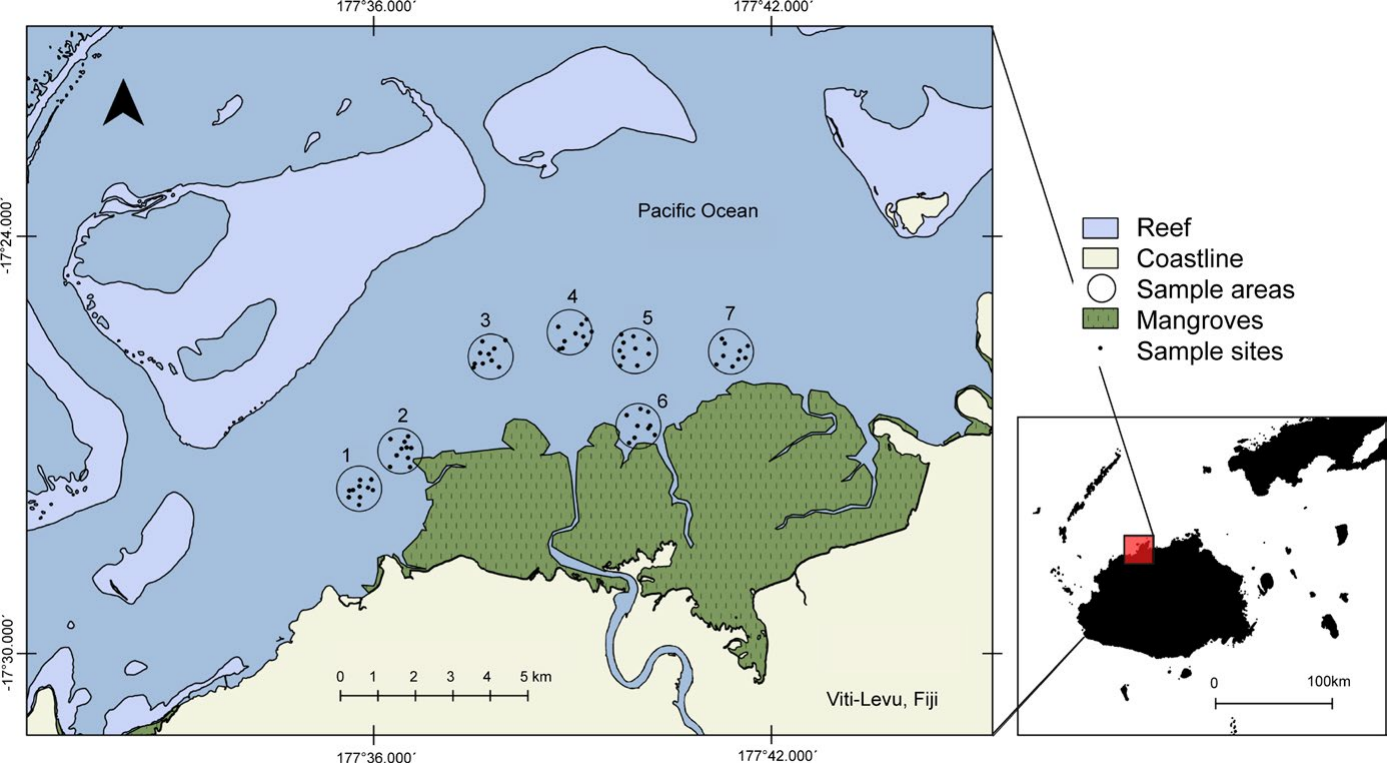
The Ba Estuary in northern Viti Levu. Circles are sampling areas 1–7; black dots denote sampling sites within sampling areas

The study was conducted in a shallow bay environment (depth < 15 m) in north‐western Viti Levu, the main island of the Republic of Fiji (Figure 1). The sampled area around the Ba River mouth is part of a larger bay that is sheltered from the open sea by patches of fringing reefs and from the mainland by mangroves. The sea bottom predominantly consists of muddy substrate and seagrass beds. The area is under strong tidal influence, with a tidal range of approximately 2 m (http://www.tide-forecast.com, 2016). There is activity by artisanal and subsistence fishermen from surrounding villages in the estuary. While sharks are by tradition not explicitly targeted in fishing operations, they regularly occur as low‐value bycatch.

### 2.2 Sampling methods

Over 6 days in November 2015, a pilot shark‐fishing survey was conducted, informed by participatory mapping with local fishermen who could indicate spots where they had previously caught sharks. The pilot study consisted of 26 gillnet deployments without a clear spatial sampling scheme in order to test the sampling methodology and procedure, and to identify suitable areas for sampling within the Ba Estuary. Deployments were conducted between 16:00 and 02:00, at varying tides and with checking intervals of 15–25 min. Total gillnet soak time of the pilot survey was 30.2 hr, during which a total of 12 sharks where caught. The catch was comprised of nine *C. limbatus* (65.5 ± 3.8 cm; seven males, two females; umbilical scar condition: five open, three semihealed, one healed) and three *S. lewini* (51.9 ± 0.7 cm; one male, two females; umbilical scar condition: one open, two semihealed). These sharks were not included in any further analyses.

Based on the results of the pilot survey, seven 1.13 km^2^ circular sampling areas in the immediate vicinity of the river mouth were selected. Each sampling area featured contrasting environmental features (e.g., depth, distance to mangroves, turbidity) and overlapped with areas that local fishermen identified as having higher shark abundances. Sampling areas 1–6 contained 10 sites each, and area 7 contained nine sampling sites (Figure 1).

The main shark‐fishing survey was conducted on 26 days from December 2015 to April 2016. Bottom‐set gillnets and longlines were set at depths ranging from 1 to 15 m in the seven sampling areas, with a total of 73 and 30 deployments, respectively. All deployments were carried out between 18:00 and 02:00 from a 7 m fiberglass boat with a 40 HP engine. Two assistants and a captain were present at all times. Bait used on longlines consisted predominantly of Indian mackerel (*Rastrelliger kanagurta*), and occasionally of red snapper (*Lutjanus argentimaculatus*), squid (*Loligo* sp.), and mullet (*Mugil cephalus*). Up to two gillnets (100 m length and 3 m width, ~10 cm mesh size) were deployed simultaneously with a soak time of 1–6 hr. To minimize animal stress and mortality, gillnets were regularly checked in intervals of 15–25 min. When feasible, a longline (75 m) with 27 hooks was additionally deployed at the same sampling sites to assess potential catch differences attributed to gear selectivity. Distance between gangions attached to the floater line varied from 2.4 to 2.8 m. Gangion length ranged between 0.6 and 3 m, with the last 0.5 m consisting of 1.5 mm steel wire and a baited 13″ circle hook. In total, fishing effort ranged from 6 to 10.36 hr (longline) and 15 to 23.08 hr (gillnet) per sampling area. Total soak time of gillnet and longline deployments varied from 30 min to 6 hr, and from 45 min to 3 hr, respectively. Sampling effort was intended to be uniformly distributed among the seven sampling areas and ranged from 24.5 to 33.3 hr/area (mean: 28 ± 3 hr/area).

### 2.3 Shark handling

All captured sharks were sexed and tagged with an internal Passive Integrated Transponder (Beijing KingDoes RFID Technologies Co., Ltd., China), as well as an external nylon spaghetti tag (Hallprint Pty. Ltd., Victor Harbor, Australia) below the first dorsal fin. Sharks were examined for umbilical scar condition (open, semihealed, healed) and measured (see Supporting Information Appendix S1 for example of open scar). Measurements were taken by placing the shark laterally on a 10 cm wide wooden board with measuring tapes attached to either side. Precaudal and fork length were read at the lower tape, while stretch‐total‐length was read from the upper tape by stretching the upper lobe of the caudal fin. If captured in a gillnet, the vertical position of the shark was classified as either being caught in the lower third (bottom: 0–1 m), the middle third (middle: 1–2 m) or upper third (top: 2–3 m) of the net. Bycatch was also recorded for each deployment. Additionally, a fin clip (ca. 0.2 cm^3^) was taken from the pelvic fin of the shark and stored in 96% ethanol for subsequent DNA barcoding (see Supporting Information Appendix S2), in order to confirm visual species identification.

### 2.4 Environmental data

To determine differences in abiotic conditions between sampling sites, and to characterize their influence on shark abundance, we measured a variety of environmental parameters selected in accordance with previous studies on drivers of habitat selection of juvenile sharks (Yates, Heupel, Tobin, & Simpfendorfer, 2015), including tide, which may also affect shark movement (Ackerman, Kondratieff, Matern, & Cech, 2000; Wetherbee, Gruber, & Rosa, 2007). Depth was recorded at the beginning and end of each gear deployment using a weighted rope and taken as the mean of both measurements. To measure turbidity, a Secchi disk was lowered in the water column until it became indistinguishable. In case of darkness, a headlight (LiteXpress liberty 120 sensor) was used to assist in determining depth. Salinity (PSU), and sea surface temperature (°C), were measured using a Manta 2 (Eureka to Water Probes, http://www.waterprobes.com). Furthermore, tide was assessed and categorized into either (a) incoming or high, or (b) outgoing or low. Geographic coordinates were determined using a Garmin Etrex 40 at the beginning and ending locations of a catch event. Distance of sampling site to mangroves (km) were calculated in QGIS 2.14.3 (Essen, http://www.qgis.org/de/site/forusers/download.html) using the distance matrix tool to measure a straight line from each sampling site to the nearest mangrove polygon. Before all executions, the coordinate system was set to EPSG:3141, Fiji 1956/UTM zone 60S. Due to logistical constraints, a complete set of environmental parameters could only be measured in 67 of the 103 deployments.

### 2.5 Analysis

We supply a descriptive analysis of species abundances in relation to sampling site and month, and of shark biodata (sex, length, umbilical scar condition). Furthermore, we statistically compared the shark catch per unit effort (CPUE) per deployment between sampling areas with a Kruskal–Wallis test, due to the non‐normal error structure of the CPUE data. For post hoc pairwise comparisons between areas, we used the non‐parametric multiple comparisons procedures provided in the R package *nparcomp* (Konietschke, Placzek, Schaarschmidt, & Hothorn, 2015). This procedure corrects for multiple hypotheses testing via multiple contrast tests and not via adjustment of significance cut‐offs (like Bonferroni correction), such that conventional levels of significance (*α* = 0.05) can be maintained without increasing the risk of Type I errors. The simultaneous two‐sided confidence intervals and *p*‐values were calculated with Tukey‐type contrasts and multivariate *t*‐distributions.

For each species, we assessed tendencies of vertical distribution in the water column by analyzing gillnet position at capture (lower, middle, or upper third, see Section 2.3) with ordinal logistic models (R package *ordinal*, Christensen, 2015), treating net positions as ordered categories.

Species‐specific time trends in umbilical scar condition over the study period (138 days from first to last deployment) were analyzed using univariate ordinal logistic models, where open, semihealed, and healed condition were treated as ordered categories that represent degree of healing. Linear models were used to analyze species‐specific time trends in length.

Finally, we assessed the association of shark abundance with environmental parameters using zero‐inflated Poisson (ZIP) models (R package *pscl*, Jackman, 2011), due to many excess zeros in the catch data (59 of 103 deployments yielded no shark catch). As environmental parameters were not measured for all deployments, models were based on the subset of *n* = 67 observations. Turbidity was excluded as a predictor, as in 13 cases of measurement the Secchi disk reached to the seafloor (we decided not to exclude these cases from analyses, but rather turbidity as a predictor, in order to maintain the already confined sample). The remaining variables—temperature, salinity, depth, distance to mangroves, and tide—were not strongly correlated with each other (all Pearson *r* < 0.3; Supporting Information Appendix S3) and thus were suitable for simultaneous inclusion in the full model. All models also included the log‐transformed effort in minutes as an offset variable. For ecological inference, we selected the best‐performing models based on AIC. We chose this information theoretic approach to assess the relevant importance of different models and predictors because of the rather exploratory nature of the study. The models with the highest predictive accuracy were selected separately for each species based on the lowest AIC values from all possible combinations of predictors. One of the sampled species, *Sphyrna mokarran*, had insufficient abundance in the reduced dataset and was excluded from ZIP analysis.

### 2.6 Interviews

A total of nine interviews were conducted with fishermen who use inshore and offshore areas around the Ba River mouth, and who inhabit the coast of the estuary. Interview information on shark occurrence was collected following the methods of Rasalato et al. (2010) and Glaus et al. (2015). Fishermen were either previously identified and approached after acquiring the consent of the headman of the respective village, or directly designated by the headman himself. Interviewees’ oral consent was obtained prior to each interview, and fishermen were informed about the project and the purpose of the survey. All interviews were conducted on a voluntary basis and anonymity and confidential treatment of all obtained data was explicitly assured. A local Fijian translator who was fluent in English and Fijian (Bauan dialect) was present at all times and assisted whenever necessary. During the semi‐structured interviews (Supporting Information Appendix S4), a visual identification poster of common inshore and offshore elasmobranch species (http://fijisharkcount.com/the-activity/all-materials/id-posters) was used to confirm species recognition. Information was collected concerning shark species occurrence, history of shark abundance over the last 15 years, and where sharks are frequently caught by operating fishermen, as a proxy for preferred habitat types. Types of fishing gear used, as well as targeting and utilization of sharks, were also assessed.

## 3 RESULTS

### 3.1 Catch composition

**Figure 2:**
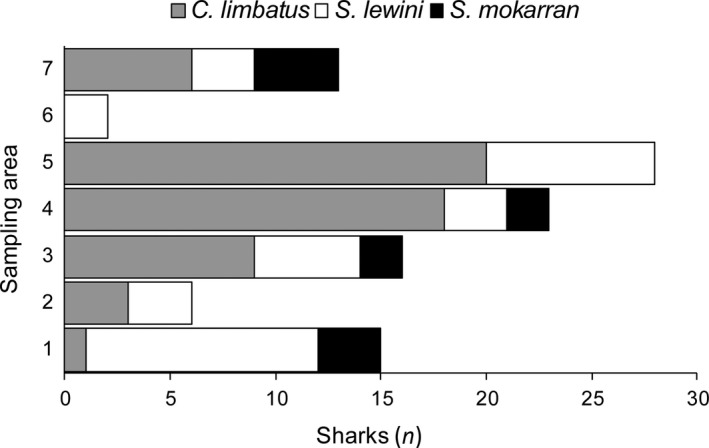
Species‐specific shark catches per sampling area

A total of 103 gear deployments were conducted. Gillnets (*n* = 73) and longlines (*n* = 30) were deployed in the seven selected sampling areas in the Ba Estuary, totalling 196.13 hr of fishing effort (Table 1) and resulting in 103 shark captures (*Carcharhinus limbatus: n* = 57, *Sphyrna lewini*:* n* = 35 and *Sphyrna mokarran n* = 11*;* see Figure 2). No sharks were recaptured during the study period.

**Table 1:** Overview of number of sharks caught per sampling area, the corresponding longline and gillnet effort and the resulting overall Catch per Unit Effort (CPUE) for each gear type and area (sharks gear^−1^ hr^−1^)

Sampling area	Total sharks caught	Sharks caught in gillnet	Gillnet hours (shots)	CPUE gillnet	Sharks caught on longline	Longline hours (shots)	CPUE longline	Total time (hr)	Pooled CPUE
1	15	13	18 (9)	0.72	2	10 (5)	0.2	28	0.54
2	6	4	19.2 (12)	0.21	2	8.1 (4)	0.25	27.3	0.22
3	16	15	23.08 (14)	0.65	1	10.22 (6)	0.1	33.3	0.48
4	23	16	18.5 (11)	0.86	7	6 (3)	1.17	24.5	0.94
5	28	25	23 (9)	1.09	3	7 (3)	0.43	30	0.93
6	2	2	17.67 (11)	0.11	0	10.36 (5)	0.00	28.03	0.07
7	13	8	15 (7)	0.53	5	10 (4)	0.5	25	0.52
1–7	103	83	134.45 (73)	0.62	20	61.68 (30)	0.32	196.13	0.53

Visual species identification could be confirmed using DNA barcoding for all 100 individuals for which a fin clip was stored. Thirty‐four sequences were positively identified as *C. limbatus* (100% bootstrap support), 30 sequences as *S. lewini* (100% bootstrap support), and six sequences as *S. mokarran* (100% bootstrap support; see Supporting Information Appendix S2 for parsimonious tree).

### 3.2 Comparison by area

Pooled CPUE per sampling area ranged between 0.11 sharks/hr (sampling area 6) and 1.09 sharks/hr (sampling area 5) for gillnet deployments, and between 0 sharks/hr (sampling area 6) and 1.17 sharks/hr (sampling area 4) for longline deployments (Table 1).

Highest monthly CPUE for gillnets was recorded in December (1.22 hr^−1^), while surveys in March had the lowest CPUE (0.25 hr^−1^; see Supporting Information Appendix S5). Longline deployments were only conducted between January and March, with the highest CPUE recorded in January (0.43 hr^−1^) and the lowest in March, when there were no shark catches at all. Gillnet CPUE was higher compared to longline CPUE in all months.

Overall CPUE varied significantly across sampling areas (Kruskal–Wallis test, *p* = 0.008; Table 1), and pairwise comparisons identified some distinct differences in CPUE between individual sampling areas (Table 2). Specifically, CPUE was higher in sampling areas 4 and 7 than in sampling area 6 (*p* < 0.05), and tended to be higher in sampling area 4 than in sampling area 2 (*p* < 0.1). Although sampling area 5 had a mean CPUE even slightly above that of sampling area 4, catch variability was almost twice as high in sampling area 5 compared to sampling area 4.

**Table 2:** Multiplicity adjusted p‐values (see section 2.5) for pairwise comparisons of CPUE between areas (all shark species combined). Bold p‐values are < 0.05. Sample size in the diagonal refers to number of deployments in the respective sampling area

Sampling area	1	2	3	4	5	6	7
1	*n* = 14						
2	0.70	*n* = 16					
3	1.00	0.95	*n* = 20				
4	0.87	0.07	0.47	*n* = 14			
5	1.00	0.91	1.00	0.96	*n* = 12		
6	0.18	0.85	0.27	**0.01**	0.38	*n* = 16	
7	1.00	0.13	0.77	0.97	0.98	**0.01**	*n* = 11

Catch composition and abundance varied in each sampling area, ranging from 28 sharks in sampling area 5 to only two sharks in sampling area 6 (Figure 2). Highest abundances of sharks (i.e., at or above median) were found in sampling areas 5 (*n* = 28), 4 (*n* = 23), 3 (*n* = 16) and 1 (*n* = 15), whereas lowest abundances were found in sampling areas 7 (*n* = 13), 2 (*n* = 6) and 6 (*n* = 2; see also Table 1). In sampling area 6, only two *S. lewini* and no other sharks were captured. *C. limbatus* dominated the shark catch in sampling areas 4 and 5 (Figure 2). In contrast, *S. lewini* was most abundant in sampling area 1 (*n* = 11), where only one *C. limbatus* individual was caught. In addition to sharks, one eagle ray (*Myliobatidae* spp.), 35 teleost species, and two crustacean species were caught as bycatch across all deployments (see Supporting Information Appendix S6). Catchability was similar between gears for *C. limbatus* and *S. mokarran* (Table 3). However, *S. lewini* was exclusively caught with gillnets.

**Table 3:** CPUE pooled across deployments by species and gear

Species	CPUE with gillnet (hr^−1^)	CPUE with longline (hr^−1^)
*Carcharhinus limbatus*	0.27	0.31
*Sphyrna lewini*	0.25	0.00
*Sphyrna mokarran*	0.06	0.05

### 3.3 Vertical net positions

**Figure 3:**
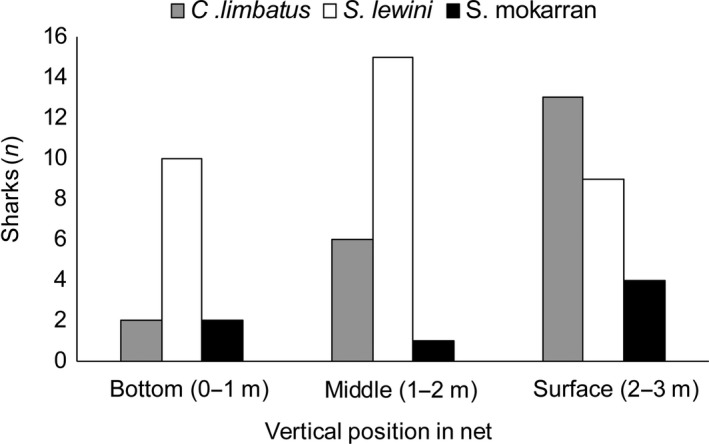
Frequency of positions of *Sphyrna lewini* (*n* = 34), *Carcharhinus limbatus* (*n* = 25) and *Sphyrna mokarran* (*n* = 7) in the gillnet

For 62 of 103 captured sharks, capture position along the vertical length of the gillnet was documented and subsequently used to explore potential partitioning of species in the water column. As also indicated by Figure 3, juvenile C. limbatus were more frequently caught in the higher positions of the net (that is, closer to the surface), as compared to juvenile S. lewini (ordinal logistic model, p = 0.01; see Supporting Information Appendix S7). Most individuals of C. limbatus were caught within the top third of the net (62 %). No difference in vertical occurrence was observed between *S. lewini* and S. *mokarran* (p = 0.29).

### 3.4 Biological shark data

**Figure 4:**
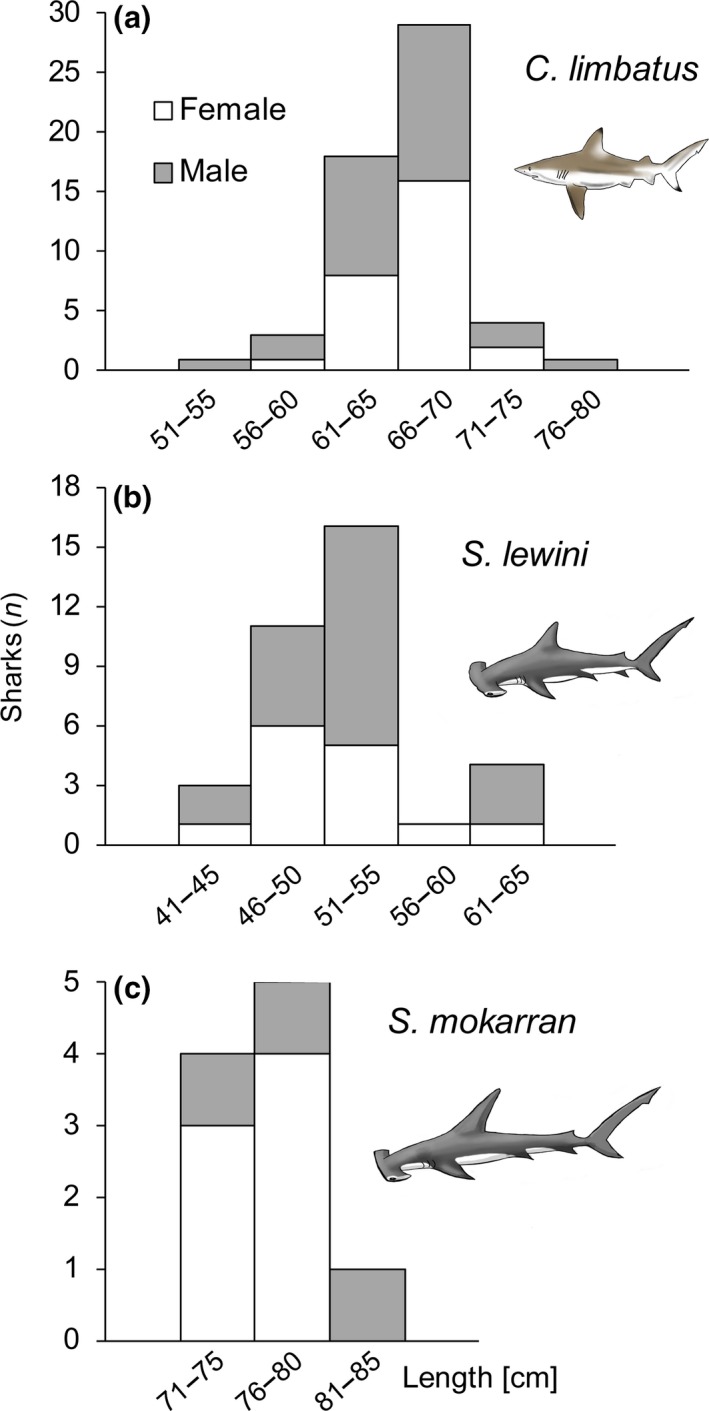
Length frequencies for (a) *Carcharhinus limbatus* (*n* = 56), (b) *Sphyrna lewini* (*n* = 35) and (c) *Sphyrna mokarran* (*n* = 10). Gray bars depict male, white bars female

Of the 103 sharks, 52 were males, 49 females, and two could not be sexed due to damage to the sharks inflicted by predatory bites while in the gillnet (Table 4). When all three species are combined, 46% of sharks captured were found to have an open umbilical scar (*n* = 47), 22% were classified as semihealed (*n* = 23), 30% as healed (*n* = 31), with the remaining two individuals being unidentifiable due to the aforementioned damage. For statistics on umbilical scar condition and length by species, see Table 4. Length distributions (Figure 4) differed significantly between all pairs of species (Kolmogorov–Smirnov tests, all *p* < 0.001).

**Table 4:** Biological shark data. Lengths and scar condition of 103 sharks and the respective sex ratio per species

	*Carcharhinus limbatus* (57)	*Sphyrna lewini* (35)	*Sphyrna mokarran* (11)	Total (103)
Open umbilical scar (%)	36 (63%)	11 (31%)	0 (0%)	47 (46%)
Semihealed umbilical scar (%)	10 (18%)	10 (29%)	3 (27%)	23 (22%)
Healed umbilical scar (%)	9 (16%)	14 (40%)	7 (64%)	30 (30%)
Unidentifiable	2	0	1	3
Precaudal length mean (±*SD*) [cm]	47.9 ± 2.7	37.2 ± 2.9	54.1 ± 2.0	/
Fork length mean (±*SD*) [cm]	54.0 ± 3.2	41.6 ± 3.4	60.5 ± 2.5	/
Total stretch length mean (±*SD*) [cm]	66.5 ± 3.8	51.8 ± 4.8	77.4 ± 2.8	/
Male:female sex ratio (not identifiable)	28:28 (1)	21:14 (0)	3:7 (1)	52:49 (2)

### 3.5 Time trends in umbilical scar and length

**Figure 5:**
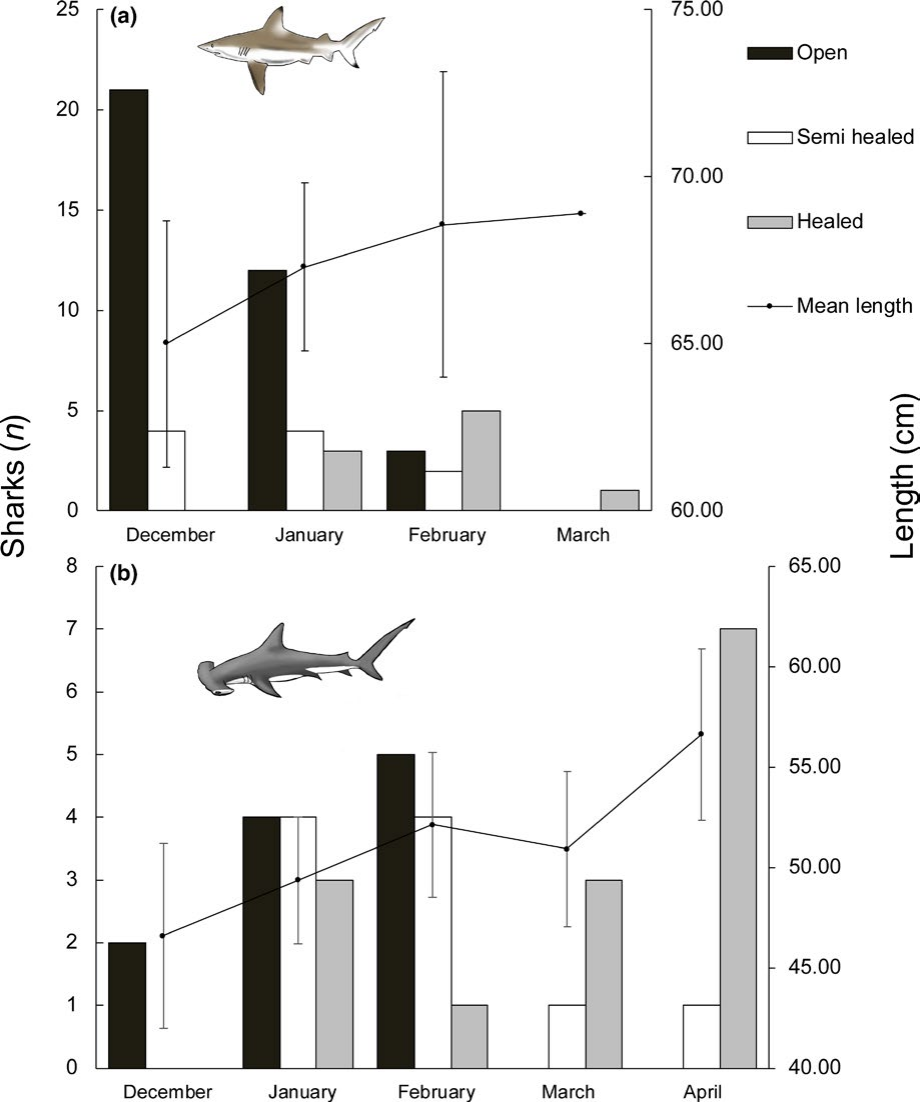
Umbilical scar condition plotted over months including mean total stretch length (in cm) for (a) *Carcharhinus limbatus* and (b) *Sphyrna lewini*. Error bars depict standard deviation

A time trend of umbilical scar condition was indicated for *C. limbatus* and *S. lewini* (Figure 5). In December 2015, most of the individuals caught exhibited open umbilical scars (*C. limbatus*:* n* = 21, 84%, *S. lewini*:* n* = 2, 100%) and no fully healed scars were encountered, while the reverse was observed in March and April. Consistent with this observation, day of the study period (from 1 to 138) significantly predicted an increase in the degree of healing of umbilical scars in ordinal logistic models (see Supporting Information Appendix S8) for both *C. limbatus* (*p* < 0.001) and *S. lewini* (*p* < 0.001). Variation in scar condition was insufficient for modelling time trends in *S. mokarran* (Table 4). In accordance with this result, linear models show that shark length increased significantly over the days of the study period for *C. limbatus* (mean increase 0.05 cm/day, *p* = 0.002; see Supporting Information Appendix S8) and *S. lewini* (mean increase 0.07 cm/day, *p* < 0.001), but not for *S. mokarran* (*p* = 0.63).

### 3.6 Environmental parameters

Among sample sites and across months, sea surface temperature varied from 29.1 to 32.5°C, and salinity varied between 27.2 and 44.6. Extreme salinity values >40 were observed in four of seven sampling areas and at sampling sites that had significantly lower depths (mean: 2.25 m) than all other sampling sites (3.54 m; Wech's *t* test, *t* = 3.0, *df* = 55.4, *p* = 0.004), thus probably a result of little mixing and strong evaporation at those sites. Depth ranged from 1.1 to 14.7 m, and distance to mangroves ranged from 0.15 to 2.6 km. Table 5 summarizes all parameters by sampling site.

**Table 5:** Summary of the environmental parameters of sampling areas 1–7

Sampling area	Measurements (*n*)	Temperature (°C)	Salinity (PSU)	Secchi depth (m)	Depth (m)	Distance to Mangroves (km)	Tide (1 = incoming or high)
1	10	29.9–31.3 (30.6, 0.5)	34.2–34.9 (34.6, 0.2)	1.5–3.5 (2.0, 0.7)	1.9–4.7 (3.2, 0.8)	1.21–1.87 (1.55, 0.22)	0.50
2	9	29.5–30.9 (30.2, 0.5)	31.4–34.9 (33.7, 1.4)	1.4–3.0 (2.2, 0.5)	2.3–6.2 (3.6, 1.3)	0.16–0.81 (0.48, 0.20)	0.33
3	10	30.4–31.9 (31.3, 0.4)	27.2–43.4 (35.2, 4.7)	0.0–2.3 (1.1, 0.7)	1.1–13.4 (4.2, 4.0)	1.43–2.23 (1.84, 0.23)	0.60
4	10	29.4–31.6 (30.8, 0.7)	33.1–44.6 (38.4, 4.4)	0.8–2.5 (1.5, 0.6)	1.4–5.0 (3.1, 1.2)	2.08–2.78 (2.40, 0.24)	0.40
5	10	29.7–32.5 (30.7, 0.8)	30.9–43.1 (36.5, 3.7)	1.0–2.5 (1.5, 0.5)	1.1–14.7 (3.7, 4.0)	1.57–2.42 (2.03, 0.28)	0.30
6	10	29.1–31.7 (30.8, 1.0)	31.8–44.2 (38.5, 5.5)	0.8–1.3 (1.1, 0.2)	1.0–3.0 (1.8, 0.6)	0.17–0.82 (0.44, 0.22)	0.40
7	8	31.2–31.9 (31.6, 0.3)	34.1–35.2 (34.7, 0.5)	1.8–2.8 (2.1, 0.4)	2.3–5.5 (3.5, 1.1)	0.44–1.23 (0.82, 0.30)	0.25

*Note*

Values indicate range and, in parentheses, mean and standard deviation. For secchi depth, 13 values were deleted, because the disk reached to the seafloor.

### 3.7 Environmental drivers of shark abundance

**Figure 6:**
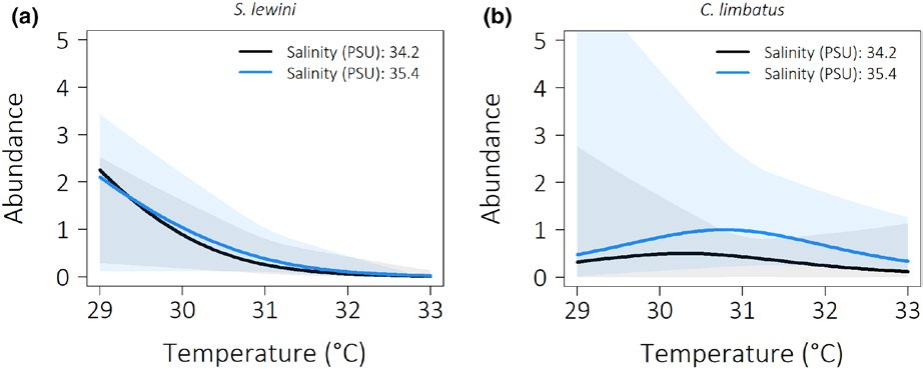
Shark abundance for the range of assessed temperatures, as predicted by the ZIP model with highest predictive accuracy for *Sphyrna lewini* (a) and *Carcharhinus limbatus* (b), respectively. The influence of salinity is visualized for the 25th (black) and 75th percentile (blue) of the sampled values, respectively, while all other variables are held at their median. Smoothened 95% confidence intervals based on bootstrapping are indicated by the colored shaded areas

For both, *S. lewini* and *C. limbatus*, temperature and salinity were the most important predictors of abundance, as they appeared in all best‐performing models (Table 6). For *C. limbatus*, there are four models that have almost identical predictive accuracy and some of them also include the predictors depth and distance to mangroves. They indicate that slightly less *C. limbatus* were caught at deeper sampling sites and at those located further from mangrove forests (Table 6). Tide did not appear to be an important covariate of abundance. Abundances of *S. lewini* and *C. limbatus* both decrease in the upper half of the temperatures range (Figure 6), although while *S. lewini* exhibits a monotonic decrease, *C. limbatus* reaches an optimum around the middle of the assessed range (ca. 31°C). The effect of salinity is negative for *S. lewini*, but positive for *C. limbatus*, as indicated by both the binomial and the Poisson estimates of the ZIP models (Table 6). Note the high uncertainty for most regions of our predictions as it is apparent in the plots (Figure 6).

**Table 6:** Zero‐inflated Poisson models with highest predictive accuracy (lowest AICs) for abundance of *Carcharhinus limbatus* and *Sphyrna lewini*

Binomial process						Poisson process						Log likelihood	AIC	ΔAIC	*w*
Intercept	Depth (m)	Temperature (°C)	Salinity (PSU)	Distance to mangroves (km)	Tide (1 = high or incoming)	Intercept	Depth (m)	Temperature (°C)	Salinity (PSU)	Distance to mangroves (km)	Tide (1 = high or incoming)				
*S. lewini*															
−6.91 (19.63)	–	1.16 (0.70)	−0.95 (0.59)	–	–	15.67 (10.44)	–	−0.39 (0.36)	−0.24 (0.09)	–	–	−44.2	100.4	0	0.26
−5.38 (21.09)	0.35 (0.30)	1.18 (0.73)	−1.05 (0.60)	–	–	17.72 (10.97)	0.04 (0.21)	−0.47 (0.40)	−0.23 (0.09)	–	–	−43.0	102.0	1.55	0.12
*C. limbatus*															
33.14 (23.69)	−0.39 (0.28)	−1.89 (0.82)	0.62 (0.41)	–	–	−7.87 (13.65)	−0.06 (0.06)	−0.92 (0.27)	0.92 (0.37)	–	–	−47.3	110.6	0	0.20
33.03 (24.75)	−0.38 (0.29)	−1.89 (0.86)	0.66 (0.44)	−0.88 (0.69)	–	−3.05 (12.50)	−0.06 (0.06)	−0.87 (0.27)	0.72 (0.35)	0.34 (0.34)	–	−45.3	110.7	0.12	0.19
17.32 (22.22)	–	−1.44 (0.77)	0.67 (0.38)	−0.85 (0.68)	–	−6.82 (12.85)	–	−0.75 (0.27)	0.71 (0.36)	0.36 (0.35)	–	−47.4	110.8	0.20	0.18
19.97 (21.00)	–	−1.56 (0.75)	0.66 (0.36)	–	–	−12.59 (14.22)	–	−0.80 (0.27)	0.94 (0.38)	–	–	−49.5	110.9	0.35	0.17

*Note*

All models within the range of two ΔAIC from the best‐performing model are shown for each species, along with their Akaike weight *w* (weight is calculated from the set of all possible models, not only from the subset of best‐fit models presented in the table). Models also contain the log‐transformed effort in minutes as an offset variable.

### 3.8 Interviews

This section reports some of the main findings from the interviews, while others will also occur in the discussion to provide context. The nine interviewed fishermen were between 34 and 68 years old. Four of the fishermen (44%) only used gillnets as fishing gear, two (22%) only used hook‐and‐line, whereas the remaining three (34%) used a combination of both gear types. Eight of the fishermen (88%) reported that they primarily target bony fish. Although all fishermen declared not to target elasmobranchs, all of them acknowledged that they regularly catch sharks as bycatch. All except one interviewee also noted that they would not like to increase their already existing shark catch. All fishermen except the two solely using hook‐and‐line use the inshore estuarine area for fishing (i.e., the area where the present study was conducted). In the case of shark bycatch, five fishermen (56%) reported caught sharks already being dead, while the remaining four (44%) stated they remained alive at the time of capture. Only two of nine fishermen (22%) described sharks as financially valuable, but only marginally. The typical price on local markets is around 2.40 USD for a bundle of four small sharks. Six fishermen (67%) reported to keep shark bycatch for personal consumption or for family and friends, while two fishermen (22%) declared they would discard them upon landing the catch. Only one fisherman reported selling them to middlemen. Even though sharks play an important traditional role in Fijian mythology and are regarded as sacred in many areas around Fiji (e.g., as the shark god Dakuwaqa; Brunnschweiler, 2010), all interviewees stated that sharks do not have any significance (e.g., cultural or bequest value) to them besides potential economic value. Five (56%) declared that they would not care if sharks disappeared from their waters, whereas four (44%) stated the opposite. When asked whether they could imagine complying with a management scheme that incorporates spatiotemporal closures of their fishing grounds in the estuary (i.e., temporarily restricting fishing in certain areas), six (67%) approved such a solution under the condition that it would be beneficial for the ecosystem. The remaining three fishermen (33%) stated they would approve such an approach under the condition that the government would compensate them for the loss of their fishing ground (two of these three people proposed a price of approximately 50 USD per month).

With the visual aid of an identification poster, fishermen reported to catch hammerhead sharks (*Sphyrna lewini* or *Sphyrna mokarran*), blacktip sharks (*Carcharhinus limbatus*), gray reef sharks (*Carcharhinus amblyrhinchos*), nurse sharks (*Nebrius ferrugineus*), whitetip reef sharks (*Trianedodon melanopterus*), and bull sharks (*Carcharhinus leucas*). Six interviewees (67%) reported that they mostly caught hammerheads. When asked about the amount of sharks caught per week and per boat, numbers varied between four to 20 sharks for the fishermen using the study area. One of the hook‐and‐line fishermen who fishes further offshore reported to capture up to 100 sharks as bycatch per trip (4–5 days) and boat (29 feet, 40 PS).

## 4 DISCUSSION

This study is the first fisheries‐independent survey on shark occurrence on the northern coast of Viti Levu, the main island of the Republic of Fiji. The results confirm the presence of juvenile blacktip sharks (*Carcharhinus limbatus*), scalloped hammerhead sharks (*Sphyrna lewini*), and great hammerhead sharks (*Sphyrna mokarran*) in the Ba Estuary. The former species is classified as “Near Threatened” (Burgess & Branstetter, 2009), and the two latter as “Endangered” (Baum et al. (2007); Denham et al. 2007), which emphasizes the important role of the area for global biodiversity. The capture of 103 juvenile individuals across 26 sampling dates from December 2015 to April 2016 indicates that the surveyed area could provide critical habitat for these coastal shark species. Whereas Rasalato et al. (2010) reported, based on local knowledge, that the Ba Estuary has a history of continuous shark presence, its species inventory, abundance, and fine‐scale distribution of sharks, as well as its function in their life‐cycle, have remained undocumented until now.

### 4.1 The Ba Estuary as a multispecies parturition ground

As exclusively juvenile sharks were encountered, it is likely that the studied nearshore environment constitutes another parturition ground in Fiji (Brown et al., 2016; Cardeñosa et al., 2017; Marie et al., 2017). Size ranges of *C. limbatus* and *S. lewini* (66 ± 4 and 52 ± 5 cm, respectively) were in accordance with size ranges of neonate and young‐of‐the‐year sharks from previously published studies (Castro, 1996; Castillo‐Géniz, Márquez‐Farias, Rodriguez de la Cruz, Cortés, & Cid del Prado, 1998; Brown et al., 2016 for *S. lewini*). For example, size of newborn *C. limbatus* range between 55 and 65 cm total length (Castillo‐Géniz et al., 1998; Castro, 1996). Although this length is slightly surpassed by individuals caught in the Ba Estuary, the current study considered total stretch length instead of total length, which would exaggerate recorded lengths during comparison. A strong indication that the Ba Estuary is used for parturition was the high proportion of sharks caught with open or semihealed umbilical scars (68%). A time frame of 5 days has been proposed for open umbilical scars to advance to semihealed status, and a further 14 days to develop from a semihealed to healed condition, based on a study conducted with captive *S. lewini* in Hawaii (Duncan & Holland, 2006).

The significant time trend in healing of scars and increase in length observed over the study period for *C. limbatus* and *S. lewini* both suggest a seasonal monotonic growth of juveniles in the Ba Estuary from December to April, with births taking place rather at the beginning of this time frame. In Australia, births of *S. lewini* were observed between September and February (Miller et al., 2013), while another study conducted in the Rewa River Delta in southern Viti Levu describes individuals with semihealed scars caught in February and March (Brown et al., 2016). Although the current study did not sample during the months of May to November, the greatest numbers of individuals with an open umbilical scar were captured in December, followed by declining numbers in January and February, and zero catches in March and April. This is broadly consistent with the time periods for parturition reported from Australia (Miller et al., 2013). Also, interviewees from local communities stated that shark abundance was highest from November to February, which supports our sampling results. Note that recapture of individuals did not occur, such that all demographic conclusions are based on a between‐individual assessment. In order to obtain a clearer picture on spatiotemporal population dynamics, we encourage larger and longer studies with a higher potential for recapture.

One additional factor that may have altered catches in March was Severe Tropical Cyclone Winston, a category 5 cyclone that hit the Republic of Fiji on 20 February 2016, with the eye passing less than 100 km north of the study area with sustained winds of 280 km/hr. Similarly, when a storm hit the Gulf Coast of Florida in 2001, 13 tagged *C. limbatus* individuals had left the area prior to the arrival of the storm and returned to the impacted area within 5–13 days (Heupel, Simpfendorfer, & Hueter, 2003).

No *S. mokarran* individuals with open umbilical scars were captured, and the species was captured in lower abundances than the other two species. This could be attributed either to overall lower abundances in the area, to other fine‐scale habitat preferences that were not covered by the sampling areas, or to utilization of the area only after parturition has occurred elsewhere. Given the low rate of encounters, its endangered status and the general paucity of ecological data, further studies are needed to investigate the importance of the Ba Estuary for *S. mokarran*.

Additionally, the main author was able to document three juvenile bull sharks (*Charcharhinus leucas*) during the study period which had been caught in gillnets by local fishermen in the Ba river several kilometres upstream the estuary. While only two could be measured (76.1 cm, 78.2 cm), fishermen confirmed fairly regular catches of small sharks of different species within the river during informal discussions and interview sessions. Bull sharks are classified as “Near Threatened” (Simpfendorfer & Burgess, 2009) and despite having been documented in other river systems of Fiji (Cardeñosa et al., 2017), no scientific record had been made in the study area before.

### 4.2 Discussion of nursery ground criteria

In addition to its likely role as a parturition ground, does the Ba Estuary also serve as a nursery ground? Nursery grounds are essential habitats for sharks, usually located in shallow inshore waters, which provide juveniles with high intake of energy and little risk of predation. The capture of individuals with healed scar conditions, especially in the later stages of our sampling time frame, suggests utilization of the Ba Estuary by juvenile sharks even after the parturition period. However, this does not yet satisfy the definition of a nursery area. According to Heupel et al. (2007), a nursery area is defined by a higher mean density of neonate or young juvenile shark abundance than in surrounding areas (criterion 1), the utilization of the area over extended periods of time (criterion 2), and the repeated use over years (criterion 3).

Our study was too locally confined to prove that shark abundance was higher in the Ba Estuary than in surrounding areas (criterion 1). The continued presence of sharks over the study period, however, fulfills criterion 2 for at least the sampled time frame. Regarding criterion 3, interviews conducted with local fishermen, additional informal talks with a range of village inhabitants, fishermen and elders, as well as the study conducted by Rasalato et al. (2010), strongly suggest that the Ba Estuary is utilized by juvenile sharks over multiple years. Distinct nursery areas of *S. lewini* and *C. limbatus* have been described (Bush & Holland, 2002; Heupel & Simpfendorfer, 2002) and both species exhibit some degree of philopatric behavior (Chapman, Feldheim, Papastamatiou, & Hueter, 2015). Anecdotal accounts by local fishermen of relatively high abundances of neonate and juvenile sharks repeatedly over years support the argument that this area is a nursery ground.

Follow‐up studies should investigate the Ba Estuary over a longer timeframe and across all seasons (wet and dry) to further substantiate these findings and systematically test all three nursery ground criteria of Heupel et al. (2007) with long‐term data. This will enable informed decision‐making about management measures, such as temporal closures or protected areas, for the maintenance of ecologically valuable shark habitats (Knip et al., 2012).

Even without final proof of whether the Ba Estuary constitutes a nursery area, Yates, Heupel, Tobin, and Simpfendorfer (2012) argue that many diverse locations might serve as important habitats to young sharks, and thus to the maintenance of populations, despite not fully meeting the three criteria of a shark nursery as defined by Heupel et al. (2007).

### 4.3 Fine‐scale distribution of species in the Ba Estuary

We found differences in total shark abundance between sampling sites, which suggests variability in the use of parts of the estuary. Sampling areas 4 and 5 yielded the highest total catches and CPUEs, while areas 2 and 6 yielded the lowest. This distribution is consistent with the reports of fishermen during the interviews. Whereas in some sampling areas (3, 4, 5) *C. limbatus* strongly dominated the catch over *S. lewini*, in others (1) the ratio was reversed.

Such differences in shark composition between areas are unlikely to be artefacts of differential gear use, because we tried to spread longline and gillnet sampling effort equally across sampling areas, which was approximately accomplished (Table 1). Furthermore, differential catchability between gears was only found for *S. lewini*, as they were exclusively caught with gillnets (Table 3). Even so, the proportion of longline effort in a sampling area does not correlate with the CPUE of *S. lewini* in an area (Pearson *r* = −0.08). Thus, we are confident that differences in *S. lewini* abundance across areas are not due to the (minor) differences in effort per gear across areas. This could be indicative of species segregation in the estuary, at least to some degree. In line with that, *C. limbatus* was almost never caught simultaneously with *S. lewini* (four cases from 46 in which at least one of both species was caught). There was also a difference in the depths at which *C. limbatus* (closer to the surface) and *S. lewini* were captured by gillnets. These instances of spatial segregation can be the result of either differential habitat selection based on physical factors (Yates et al., 2015) or direct interspecific processes like competition for space and food resources (White, Platell, & Potter, 2004). Competitive interactions (and thus selection) are theorized to occur with higher intensity within nursery areas (Heithaus, 2007). Also, the three juvenile bull sharks caught upstream by fishermen during the study period might avoid competition and predation risk by occupying freshwater that is inaccessible to other shark species (Heupel & Simpfendorfer, 2011), although this remains speculative.

There is evidence that environmental factors play some role in habitat partitioning in the Ba Estuary, as the best‐performing models for fine‐scale (i.e., between sampling sites) drivers of shark abundance in our sample predict different responses to physical factors for the two species we sampled with highest frequency, *C. limbatus* and *S. lewini*. For example, with salinities approaching the higher values of the sampled range, abundance is predicted to increase for *C. limbatus* and to decrease for *S. lewini*. Strikingly, 18 individuals of *C. limbatus* were captured in sampling area 4 that had salinity measurements as high as 40 PSU. These findings differ from the preferred salinity range of *C. limbatus* as described in a long‐term study (32 years) off the coast of Texas (Froeschke, Stunz, Sterba‐Boatwright, & Wildhaber, 2010), where they predominantly occurred in moderate salinities ranges (20–35). Osmoregulation is energy‐consuming for sharks, with the largest energy expenditure presumably required when surface to volume ratio is lowest, that is among juveniles (Heupel & Simpfendorfer, 2008). Abundance of young *S. lewini* increased with salinity in a study in the north‐eastern Gulf of Mexico, where they preferably occurred at salinities >35 (Ward‐Paige, Britten, Bethea, & Carlson, 2015).

Model estimates on environmental drivers of abundance should generally be very sensitive to the sampled range of the variable, such that differences between studies and regions are to be expected. This is obvious in the abundance predictions for the temperature range we sampled. Our models predict that abundance of *S. lewini* decreases monotonically with temperature, which contrasts the finding of Ward‐Paige et al. (2015) and Yates et al. (2015), who find an opposite effect in the Gulf of Mexico and Australia, respectively. Importantly, the temperature range they sampled (e.g., Yates et al., 2015 in Australia, minimum: <20°C) is very different to that encountered within this study (minimum: 29°C), which likely leads to the reverse scenario for the Ba Estuary.

Up to a point, warmer temperatures can induce faster growth and boost metabolic rates by increasing rates of biochemical reactions (Froeschke et al., 2010; Heupel et al., 2007). Thus, the overall high sea surface temperatures of the Ba Estuary (29–32°C) may benefit the juvenile sharks by maximizing physiological performance, as long as they do not surpass a critical threshold. Accordingly, catch rates for all shark species increased with temperatures from 20 to 33°C in a study conducted along the Texan coast, before declining again above 33°C (Froeschke et al., 2010). Given this information and based on our own models (Figure 6), the Ba Estuary might represent a habitat at, or in some parts even slightly above, the upper limit of tolerable temperatures for these sharks. Rising ocean temperatures in coastal waters, as is projected with climate change, might thus lead to altered spatial distributions or higher mortality rates (Bangley et al., 2018; Chin et al., 2010).

Turbidity can also affect habitat choice in juveniles (Yates et al., 2015), but measurements in this study were not sufficient to be included in our analyses. Turbidity is considered to facilitate predator avoidance for young sharks (Heupel et al., 2007). Other factors like prey availability can also influence habitat use (Torres, Heithaus, & Delius, 2006). However, the majority of the bycaught teleosts in our study exceeded the size of potential prey, such that we lack a proxy for prey density.

### 4.4 Management implications

Taking a social‐ecological perspective, our study offers insights for future fisheries management in the area. The Ba Estuary and its adjacent fishing grounds belong to the Votua *qoliqoli* (customary fishing ground) and is among the largest within the Republic of Fiji. It is fished by approximately 150 licensed boats, excluding the poachers that are frequently observed and reported (T.V. personal communication, 2016), and mainly with gillnets.

Strikingly, and opposed to other artisanal fisheries where sharks have high economic and consumption value (e.g., the Gulf of Mexico, Castillo‐Géniz et al., 1998), the main anthropogenic threat to sharks in the study area results from bycatch. Shark bycatch is a problem of global magnitude (Bonanomi et al., 2017). However, compared to the situation in communities with shark‐targeting fisheries, where conservationists and resource users experience conflicting interests, this has the positive implication that an agenda to reduce shark catch is not against the economic interests of fishermen in the Ba Estuary. Indeed, our interviews showed that fishermen desire to avoid catching sharks due to their low economic value, and that they would largely support spatiotemporal closures in conjunction with financial compensation schemes.

Temporal closures are not a new concept to Fijians, as their traditional *tabu* system refers to the part‐time prohibition of fishing within selections of the *qoliqoli* following events of social significance (e.g., death of a chief) to allow recovery of certain fish species and maintain overall ecosystem health (Caillaud et al., 2004). Contemporary governance approaches in Fiji often incorporate area management based on customary systems (Jupiter et al., 2017). Thus, there is strong potential for community support. Such support and understanding by the local population is crucial for the successful implementation of fisheries closures (Bennett & Dearden, 2014). Both our ecological and our fishermen‐based survey indicate suitable time frames (November to February) and areas (sampling areas 4 and 5) for such closures due to high concentrations of juveniles and potential parturition.

Importantly, closures are not a panacea to integrate biodiversity conservation and development (Adams et al., 2004), and other measures to reduce shark bycatch exist, such as gear modifications (Bonanomi et al., 2017). Ultimately, the success of any strategy will depend on whether a co‐management regime is successful in maintaining fisheries or alternative livelihoods and, at the same time, in being adjusted to the life‐histories of the local shark species. This can be especially challenging for species like *S. lewini*, which has a particularly low potential for population recovery (Branstetter, 1987; Smith et al., 1998). Both will require further research, or even experimentation with policy schemes and continued monitoring (i.e., adaptive management; Folke, Hahn, Olsson, & Norberg, 2005). If, for example, fishing restrictions result in protection of sharks but also transparent co‐benefits for fishermen through the replenishment of fished teleost stocks (Aburto‐Oropeza et al., 2011), such an intervention has good chances of being enforced and institutionalized even by the communities themselves (Ostrom, 2000).

The fact that interviewed fishermen reported additional species to occur in the estuary that we did not sample, such as whitetip reef or nurse sharks, further emphasizes the need for appropriate local conservation policies and potentially the incorporation of fishermen's catch or landing data into assessments of local shark occurrence. Interestingly, the majority of interviewees (67%) reported to mainly catch hammerhead sharks as bycatch, while our own sampling predominantly yielded blacktips (62%). This difference might be partly due to the characteristic appearance of hammerhead sharks making them more prone to be remembered, and highlights the need for complementarity of indirect (Rasalato et al., 2010) and direct shark surveys such as this study.

While there is currently no practical solution to eradicate shark bycatch, there are several possibilities to minimize it if policy and decision‐making processes incorporate scientific data into their agenda. Countries such as the Republic of Fiji, where sharks naturally occur within the national territory, have a responsibility to ensure the long‐term survival of these endangered species by adopting national management plans that support global biodiversity, such as the protection of critical habitats.

## CONFLICT OF INTEREST

Authors have no conflict of interest to declare.

## AUTHOR CONTRIBUTION

T.V., C.R., and A.D.M. designed the study. C.R. and A.D.M. wrote the project proposal and obtained funding. T.V. conducted the fieldwork and the interviews. T.V. and S.G. developed the sampling scheme, all analyses and wrote the first draft of the manuscript. All authors contributed to the write up of the final version of the manuscript. This study is part of the M.Sc. thesis of T.V., supervised by C.R. and M.Z.
