# Supplementary material for: Discovery of a multispecies shark aggregation and parturition area in the Ba Estuary, Fiji Islands
Source: Ecol Evol. 2018 Jun 25;8(14):7079–93. doi: 10.1002/ece3.4230 (PMC6065273; doi:10.1002/ece3.4230)
Supplement: Supplementary file 1 [file ECE3-8-7079-s001.docx]

SUPPLEMENTARY MATERIAL for

**Discovery of a multi-species shark aggregation and parturition area in the Ba Estuary, Fiji Islands**

Tom Vierus, Stefan R. Gehrig, Juerg M. Brunnschweiler, Kerstin Glaus, Martin Zimmer, Amandine D. Marie and Ciro Rico

Contents

[**S1: Umbilical scar example** 2](#_Toc504642409)

[**S2: DNA barcoding** 3](#_Toc504642410)

[**S3: Correlations between environmental parameters** 6](#_Toc504642411)

[**S4: Semi-structured interviews** 7](#_Toc504642412)

[**S5: Pooled CPUE across months** 11](#_Toc504642413)

[**S6: Bycatch inventory** 12](#_Toc504642414)

[**S7: Ordinal logistic model on vertical distribution in the gillnet** 14](#_Toc504642415)

[**S8: Ordinal logistic model on time trends in scar conditions and linear models on time trends in length** 15](#_Toc504642416)

# **S1: Umbilical scar example**


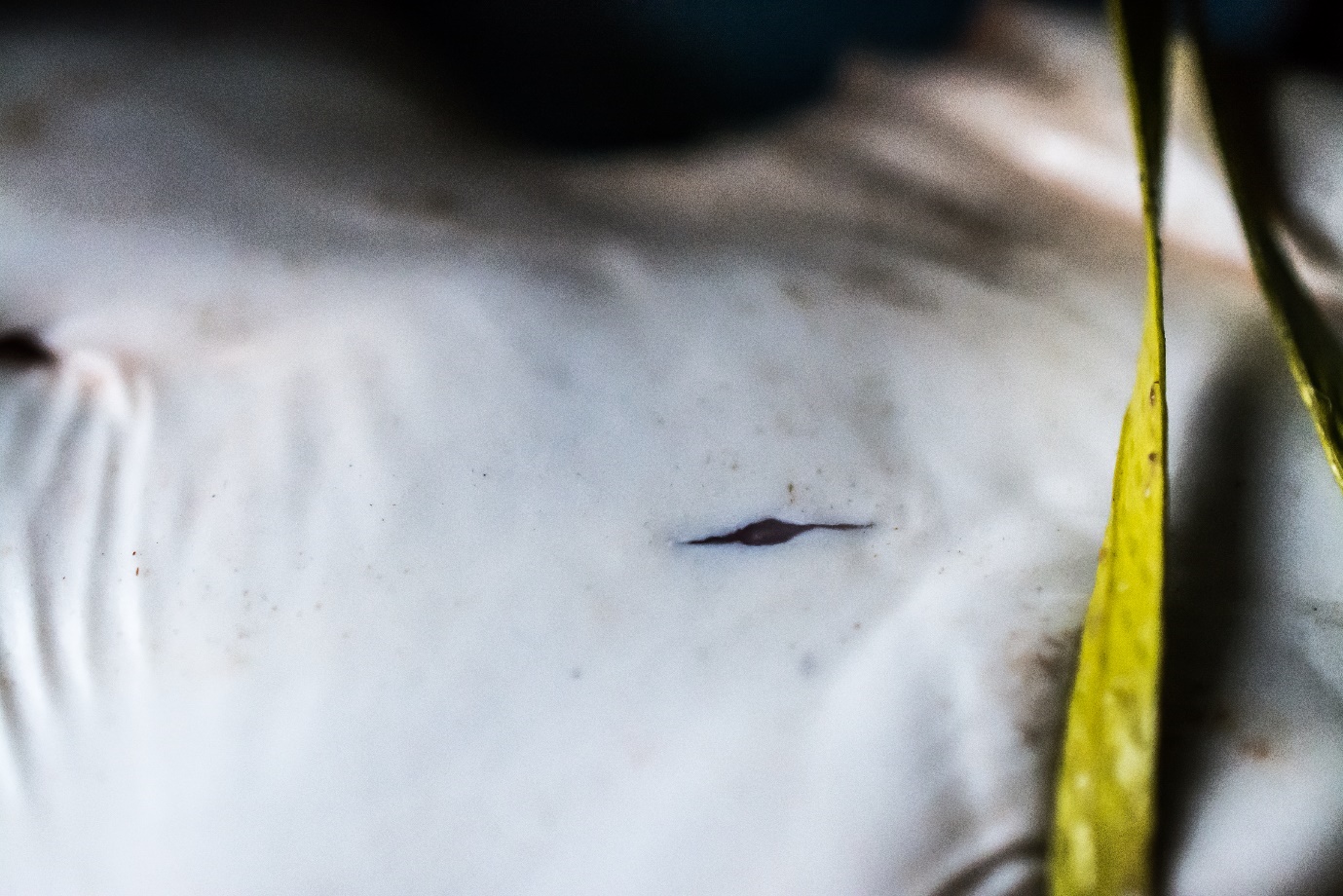


Open umbilical scar of a blacktip shark (*Carcharhinus limbatus*) captured in the study.

Photo: Tom Vierus.

# **S2: DNA barcoding**

Material & Methods:

Collected tissue samples were extracted following Aljanabi and Martinez (1997) with minor adaptations. DNA samples were amplified by PCR using the mitochondrial COI-1 primers commonly employed for DNA barcoding (FF2d and FR1d; Ivanova *et al.* 2007). DNA samples were Sanger-sequenced by Eurofins GmbH (Ebersberg, Germany) and subsequently compared with consensus BOLD sequences of all available COI sequences at the National Center for Biotechnology Information (www.ncbi.nlm.nih.gov) of the following sharks [species name (number of sequences used for the consensus)]: *Carcharhinus limbatus* (211), *Carcharhinus melanopterus* (104), *Carcharhinus tilstoni* (68), *Carcharhinus amblyrhynchos* (248), *Carcharhinus brevipinna* (272), *Carcharhinus sorrah* (184), *Carcharhinus leiodon* (28), *Carcharhinus plumbeus* (119), *Carcharhinus leucas* (152), *Carcharhinus longimanus* (140) as possible candidates for the unknown blacktip species, or *Sphyrna lewini* (269), *Sphyrna mokarran* (59), *Sphyrna zygaena* (48), *Sphyrna tiburo* (45) for the hammerhead shark samples. Available COI-sequences of the respective sharks were aligned with the ‘Clustal View Alignment’ tool (Thompson *et al.* 1994) in BioEdit and amalgamated into a consensus sequence. Clipped versions of forward reads (provided by Eurofins) were aligned with all ten consensus sequences for blacktips, and four for the hammerheads, using Mafft with the ‘–reorder’ option (Katoh *et al.* 2002). Groups of sequences without mismatches (though varying lengths) were identified and then merged into consensus sequences as described above. Subsequently, we calculated a phylogenetic tree with a neighbour-joining algorithm using the default setting in Mega 7.0 (Kumar *et al.* 2016), but managing missing data through ‘pairwise deletion’ instead of ‘complete deletion’. This approach ensured that missing data in the alignments were taken into account. Additionally, a second phylogenetic tree with bootstrap support and the exclusion of the two species, *C. leidon* and *C. tilstoni,* was calculated, as these two species do not occur within Fijian waters (*C. leiodon* in northern Indian Ocean, *C. tilstoni* in Australia).

Fin clips (ca. 0.5 – 1 cm2) were cut into smaller pieces using a scalpel, transferred in a 1.5 ml tube and 440 µl of salt extraction buffer, 44 µl of SDS (20 %) and 10 µl of Proteinase K (Analytic Jena or Geneon) was added. The samples were incubated for one hour at 60 °C followed by the addition of 300 µl 5M NaCl and centrifuged for 30 min at 10.000 G. After transferring 600 µl of the supernatant into a new 1.5 ml tube, 600 µl of ice-cold Iso-Propanol (-20 °C) was added and the samples were kept at -20 °C for 20 min before centrifuged for 20 min at 10.000 G. After discarding the supernatant, 200 µl of ice-cold EtOH (70%) was added and the samples transferred into a centrifuge for 10 min at 10.000 G. The supernatant was discarded, and the tubes dried for one hour at room temperature. The pellets were then resuspended in 100 µl TRIS-Buffer (pH 8) and stored at -20 °C.

All PCR’s had a total volume of 25 µl and included: 18.25 µl dest. H2O, 2.5 µl of (10x) Buffer B or C, 1 µl of MgCL2, 0.5 µl of dNTPs (10 mM), 0.5 µl of each primer, 0.75 µl of either Opti Taq or Color Opti Taq Polymerase (Roboklon) and 1 µl of the DNA Template. The thermocycler profile for the COI target region was set as follows: A two-minute activation of the polymerase at 95°C followed by 35 cycles of 20 sec at 95 °C, 40 sec at 50 °C, one min at 72 °C and an final extension step of ten min at 72 °C. Subsequently, PCR products were visualized on a 1 % agarose gel and investigated for successful amplification of 681 bp under UV transillumination. Five µl of each PCR product was transferred into a new 0.5 ml tube before adding two µl of ExoSAP-IT (Affymetrix) and subsequently heated to 37 °C for 15 min to digest single-stranded DNA and remove phosphate groups, before sending them for Sanger sequencing (Eurofins GmbH, Ebersberg, Germany). Sequences were then manually edited in Bioedit 7.2.5 (Hall 1999).

Results:


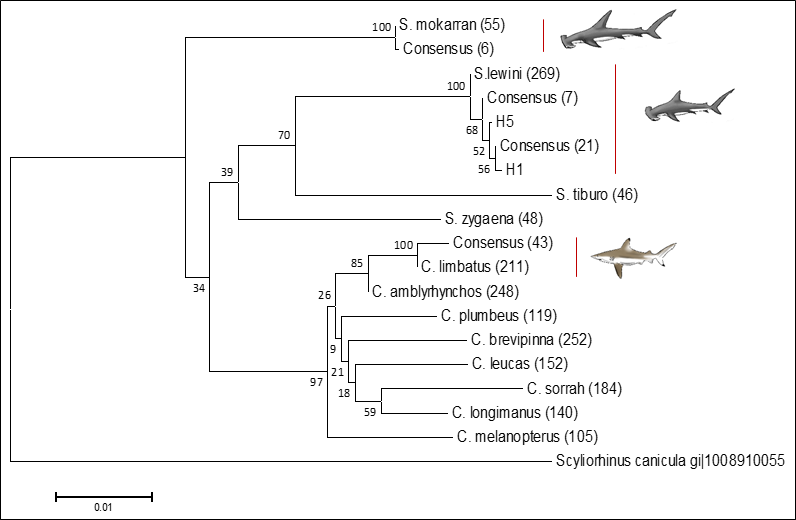


Neighbor-Joining phylogenetic tree calculated with sequenced COI forward reads and available COI BOLD sequences of respective possible sharks from the National Center for Biotechnology Information. The tree is rooted with the small-spotted catshark *Scyliorhinus canicula.* Node support after 500 bootstrap replications is indicated. Support of 100 % was only found in nodes including the sequenced forward reads (*S. mokarran, S. lewini, C. limbatus*). Numbers in brackets report the number of sequences used for the consensus sequences. Scale bar indicates nucleotide substitutions per site.

References:

Aljanabi, S.M., Martinez, I., 1997. Universal and rapid salt-extraction of high quality genomic DNA for PCR-based techniques. Nucleic Acids Res. 25, 4692–4693. doi:10.1093/nar/25.22.4692

Hall, T. A. (1999). BioEdit: a user-friendly biological sequence alignment editor and analysis program for Windows 95/98/NT. *Nucleic Acids Symp. Ser.* 41, 95-98

Ivanova, N. V., Zemlak, T.S., Hanner, R.H., Hebert, P.D.N., 2007. Universal primer cocktails for fish DNA barcoding. Mol. Ecol. Notes 7, 544–548. doi:10.1111/j.1471-8286.2007.01748.x

Katoh, K., Misawa, K., Kuma, K., Miyata, T., 2002. MAFFT: a novel method for rapid multiple sequence alignment based on fast Fourier transform. Nucleic Acids Res. 30, 3059–3066. doi:10.1093/nar/gkf436

Kumar, S., Stecher, G., Tamura, K., 2016. MEGA7: Molecular Evolutionary Genetics Analysis version 7.0 for bigger datasets. Mol. Biol. Evol. 33, msw054. doi:10.1093/molbev/msw054

Thompson, J.D., Higgins, D.G., Gibson, T.J., 1994. CLUSTAL W: Improving the sensitivity of progressive multiple sequence alignment through sequence weighting, position-specific gap penalties and weight matrix choice. Nucleic Acids Res. 22, 4673–4680. doi:10.1093/nar/22.22.4673

# **S3: Correlations between environmental parameters**

|  | Temperature (°C) | Salinity (PSU) | Secchi depth (m) | Depth (m) | Distance mangroves (km) | Tide (1 = high or incoming) |
| --- | --- | --- | --- | --- | --- | --- |
| Temperature (°C) | 1 | -0.01 | 0.08 | -0.08 | 0.03 | -0.20 |
| Salinity (PSE) | -0.01 | 1 | -0.17 | -0.16 | 0.09 | 0.02 |
| Secchi depth (m) | 0.08 | -0.17 | 1 | 0.36 | 0.11 | -0.17 |
| Depth (m) | -0.08 | -0.16 | 0.36 | 1 | 0.24 | -0.19 |
| Distance mangroves (km) | 0.03 | 0.09 | 0.11 | 0.24 | 1 | 0.05 |
| Tide (1 = high or incoming) | -0.20 | 0.20 | 0.17 | -0.19 | 0.05 | 1 |

# **S4: Semi-structured interviews**

I am Tom Vierus, a student from Germany, and I am currently doing my master thesis research with the University of the South Pacific. I am part of a research team that assesses the situation of several in Fiji and aims to establish a national management plan as a final outcome. To achieve this the major rivers on Viti Levu and Vanua Levu will be assessed in regard of their role as a nursery area for juvenile sharks.

Therefore, I would like to ask your permission and time to answer the following questions. It should take less than 60 minutes.

If you do not wish to answer certain questions, feel free to do so or stop the questionnaire at any time. If you wish, you can write down the answers either in English or in Bauan Fiji.

If you have any questions or concerns about any parts of this research, please contact us at any time.

ZMT Bremen (Germany): Tom Vierus, email: tom@vierus.de

USP, Suva (Fiji): Prof. Dr. Ciro Rico, email: ciro.rico@usp.ac.fj

Date Interviewer

Village Translator

Male/Female Oral Consent

1. General shark & fishing questions

1.1. What do you usually catch? (Fish, Crabs, Prawns, Beche-de-mer, Other___)

1.2. How many times do you go fishing per week? (Every day, 5-6 times, Less than 5 times, Other ___)

1.3. When do you usually go fishing? (During the day, During the night, Both, Other ____)

1.4. How big is your boat (feet/meters) and which engine do you use? Size: _________ Engine: ________

1.5. Which fishing gear types(s) do you use? Multiple choices possible! (Gill nets, Hook & Line, Spearfishing, Drumlines/Longlines, Other ____)

1.6. How many people do you go fishing with?

1.7. Where do you usually fish? (Inshore, Offshore, Coral Reef, Rivers, Other ___)

1.8. Do you catch sharks?

1.9. If yes, do you target them or catch them as bycatch?

1.10. If you had sharks as bycatch, are they usually dead or alive?

1.11. If you had shark bycatch, what do you normally do with it? (Discard, Keep it for personal consumption, Sell on market, Other ___)

1.12. If targeted, do you target specific sharks and why?

1.13. If targeted, where do you fish for sharks? (Inshore, Offshore, Coral Reef, Rivers, Other ___)

1.14. Where do you catch most of the sharks? (Inshore, Offshore, Coral Reef, Rivers, Other ___)

1.15. Which fishing gear type(s) is most effective to catch sharks?

1.16. How many sharks do you approximately catch per week and boat?

1.17. Would you like to catch more sharks? If yes, why?

1.18. Which kinds of sharks do you catch? Please identify them on the poster!

1.19. Which one of the mentioned sharks do you catch the most?

1.20. Do you know whether sharks are protected around your fishing grounds?

1.21. If yes, how are they protected?

1.22. Is anybody checking on your fishing?

1.23. If yes, who and how often? Do you have any negative associations with sharks or have you had negative experiences with them in the past?

1.24. How would you rate the shark stocks around your fishing grounds from a scale from 1 -1 0, with 10 being very high shark abundance and 1 being very scarce encounters?

1.25. Have there been changes over the last 15 years in shark abundance?

1.26. If yes, how?

1.27. What do you think is the reason for the change?

1.28. What do you think will happen in future? How will the situation develop?

2. Scalloped hammerheads shark questions


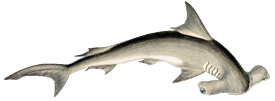


2.1. This is a picture of a scalloped hammerhead shark. Do you know this animal and if yes, how? Chose all applicable options!

(1) No, I don`t know this animal

(2) I know from others that they exist here, but haven`t seen them

(3) I have seen them personally on the market

(4) I have seen them personally in the waters

(5) I have fished them from the waters

2.2. What are the local names for the hammerhead shark?

2.3. Is the hammerhead shark of any financial value to you? (Yes, No)

2.4. If yes, how much is one individual worth or how much is a kg sold for?

2.5. Please circle the areas where you catch most of the sharks!


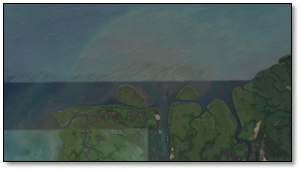


2.7. If you catch them, would you like to catch more hammerhead sharks? (Yes, No, I don’t care)

2.8. Please give reasons for your answer. (No, because .. , Yes, because .. )

2.9. Which sizes do you catch? 1: smaller than one meter, 2: bigger than 1m, 3: both

2.10. Is the hammerhead shark of any significance to you other than economically?

2.11. If yes, please explain!

2.12. Are there a lot of hammerhead sharks in your fishing area?

2.13. Has their abundance/occurrence/catches changed over the last ten years?

2.14. If yes, how?

2.15. Would you mind if the hammerhead shark disappeared from your waters in future? (Yes, No, I don’t care)

2.16. Would you be willing to include parts of your fishing grounds for conservation-oriented management, e.g. refrain from fishing there during high season of shark births (~December-February)?

(1) Yes, if it is beneficial for the natural system

(2) Yes, but only if I am compensated by the government/province, e.g. by ________ per week

(3) No, I want to continue using the entire area

3. Personal information

3.1. What is your home village?

3.2. How old are you?

3.3. For how many years have you been fishing?

3.4. How many boats/fishermen are currently fishing in and around the Ba river?

3.5. Where are they coming from?

3.6. Is fishing your main occupation? Do you have or did you have other occupations?

3.7. How much income do you get per week from fishing, fishing related to sharks in general and related to only hammerheads and are there any other sources of income in your household? If yes, which, by whom and how many? (please state the source, by whom it is earned and the amount per week)

# **S5: Pooled CPUE across months**


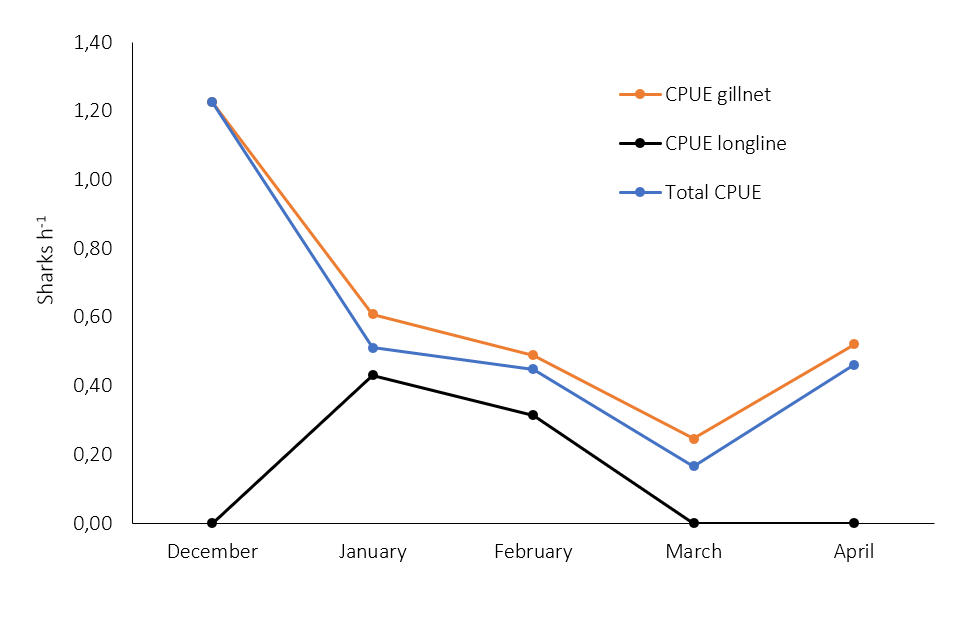


Monthly CPUE pooled across deployments. Sharks captured per hour and with gillnet (orange), longline and with both gears combined (blue).

# **S6: Bycatch inventory**

Fijian names as described by fishermen and English translation. Note that names can vary among areas in Fiji and some could not be translated (*NA*). The most specific taxonomic classification for each group varies, because species-level identification did not take place consistently in the field.

| **Fijian name (number of catches)** | **English** | **Taxon** |
| --- | --- | --- |
| Baba (8) | Needlefish | *Belonidae* |
| Barrakuda (5) | Barracuda | *Sphyraenidae* |
| Bogi (4) | *NA* | *NA* |
| Bunabu (1) | Chub | *Kyphosidae* |
| Busa (2) | Halfbeaks | *Hemiramphida* |
| Chowa (8) | *NA* | *NA* |
| Crab (6) | Crab | *Brachyura* |
| Damu (33) | Red Snapper | *Lutjanidae* |
| Moli (1) | *NA* | *NA* |
| Nightfish (1) | Nightfish | *NA* |
| Prawn (1) | Prawn | *Penaeus* |
| Remora (7) | Remora | *Echeneidae* |
| Sanga (12) (Saqa) | Jacks or Trevally | *Caranx* |
| Sony (185) (Soni) | *NA* | *NA* |
| Eaglye Ray (1) | Eagle Ray | *Aetobatus narinari* |
| Gitawa (Qitawa) (5) | Jarbua | *Terapon jarbua* |
| Kabatia (1) | Emperor | *Lethrinidae* |
| Kake (3) | Snapper | *Lutjanidae* |
| Kalo (2) | *NA* | *NA* |
| Ki (4) | Ponyfish | *Leiognathidae* |
| Lawe (Labe) (1) | Wrasse | *Labridae* |
| Malaya (1) | Tilapia | *Tilapia* |
| Mullet (10) | Mullet | *Mugilidae* |
| Nunga Nunga (Nuqa) (3) | Rabbitfish | *Siganidae* |
| Salala (44) | Milkfish | *Chanos chanos* |
| Sanya (3) | *NA* | *NA* |
| Tutu (30) | *NA* | *NA* |
| VanuVanu (2) | *NA* | *NA* |
| Vavula (1) | *NA* | *NA* |
| Vocia (19) | Mullet | *Mugilidae* |
| Vuna (2) | *NA* | *NA* |
| Wakua (1) | Parrotfish | *Scaridae* |
| Watuwatu (12) | *NA* | *NA* |
| Yaela (2) | *NA* | *NA* |
| Yandrava (Yadrava) (1) | *NA* | *NA* |
| Yavula (5) | *NA* | *NA* |
| Yawa (1) | *NA* | *NA* |
| Yawinda (Yawida) (1) | *NA* | *NA* |

# **S7: Ordinal logistic model on vertical distribution in the gillnet**

Reference categories: bottom, *S. lewini* (n = 62)

| Variable | Estimate | Std. Error | P-value |
| --- | --- | --- | --- |
| Intercept (middle) | -0.78 | 0.35 | - |
| Intercept (surface) | 0.92 | 0.36 | - |
| *S. mokarran* | 0.90 | 0.84 | 0.29 |
| *C. limbatus* | 1.41 | 0.55 | 0.01 |
| Log-likelihood: -62.6, Likelihood ratio test: X² = 7.21, df = 2, p = 0.027 | | | |

# **S8: Ordinal logistic model on time trends in scar conditions and linear models on time trends in length**

Ordinal logistic model on time trend of umbilical scar condition (reference category: open) for *S. lewini* (n = 35).

| Variable | Estimate | Std. Error | P-value |
| --- | --- | --- | --- |
| Intercept (semi-healed) | 1.650 | 0.807 | - |
| Intercept (healed) | 3.294 | 0.954 | - |
| Study day | 0.035 | 0.010 | < 0.001 |
| LogLikelihood: -30.94, Likelihood ratio test: X² = 14.29, df = 1, p < 0.001 | | | |

Ordinal logistic model on time trend of umbilical scar condition (reference category: open) for *C. limbatus* (n = 55).

| Variable | Estimate | Std. Error | P-value |
| --- | --- | --- | --- |
| Intercept (semi-healed) | 2.084 | 0.58 | - |
| Intercept (healed) | 3.336 | 0.69 | - |
| Study day | 0.039 | 0.011 | < 0.001 |
| LogLikelihood: -41.24, Likelihood ratio test: X² = 14.71, df = 1, p < 0.001 | | | |

Linear model on time trend of length for *S. lewini* (n = 35).

| Variable | Estimate | Std. Error | P-value |
| --- | --- | --- | --- |
| Intercept | 46.644 | 1.556 | < 0.001 |
| Study day | 0.066 | 0.018 | < 0.001 |
| R² = 0.296 | | | |

Linear model on time trend of length in cm for *C. limbatus* (n = 56).

| Variable | Estimate | Std. Error | P-value |
| --- | --- | --- | --- |
| Intercept | 64.825 | 0.700 | < 0.001 |
| Study day | 0.050 | 0.016 | 0.0023 |
| R² = 0.160 | | | |

Linear model on time trend of length in cm for *S. mokarran* (n = 10).

| Variable | Estimate | Std. Error | P-value |
| --- | --- | --- | --- |
| Intercept | 78.558 | 2.586 | < 0.001 |
| Study day | -0.022 | 0.044 | 0.631 |
| R² = 0.030 | | | |
